# Palindromic Rheumatism Improved After Bariatric Surgery: A Case Report

**DOI:** 10.7759/cureus.48938

**Published:** 2023-11-17

**Authors:** Salem K Qupp, Sanaa F Zeidan, Hazar M Ghneim, Roba S Makhlouf, Aisha M Najajrah, Aisha S Muslih, Saja J Fkheidah, Abdurrahman Y Kawasmi

**Affiliations:** 1 Medicine, Al-Quds University, Jerusalem, PSE; 2 Internal Medicine, Al-Quds University, Jerusalem, PSE

**Keywords:** rheumatic diseases, bariatric surgery, obesity, intermittent joint inflammation, rheumatoid arthritis, palindromic rheumatism

## Abstract

Palindromic rheumatism (PR) is a unique syndrome considered a prelude to rheumatoid arthritis (RA). It is characterized by recurrent, unpredictable episodes of joint inflammation and distinct clinical features. Unlike RA, PR episodes are brief and reversible, involving sudden-onset joint pain, swelling, and erythema. The exact etiology and diagnostic criteria of PR remain elusive, but it often shares autoantibodies with RA, leading patients to transition from PR to RA. The management of PR is multifaceted and empirical, involving various treatment modalities such as non-steroidal anti-inflammatory drugs (NSAIDs), colchicine, and disease-modifying anti-rheumatic drugs (DMARDs). However, the relationship between obesity and PR remains underexplored.

This case presents a 52-year-old woman, who presented to our hospital with recurrent and debilitating arthritis episodes since 2016. Initially affecting her wrists and later extending to her knees, shoulders, and back, these episodes severely impaired her daily activities. Despite a diagnosis of RA in 2019, supported by a positive rheumatoid factor (RF) test, and subsequent DMARD treatment, her symptoms persisted. In 2022, during her examinations at our hospital, the distinctive pattern of intermittent symptoms accompanied by damage-free joints was unveiled, suggesting a potential diagnosis of palindromic rheumatism.

Notably, this case highlights the potential association between obesity and PR, as the patient's decision to undergo bariatric surgery in 2022 led to substantial weight loss of over 36 kg. This weight reduction yielded remarkable improvements in her condition, resulting in reduced frequency and severity of PR attacks. As a consequence, her medication regimen was simplified, emphasizing the therapeutic role of weight management in PR. This case paves the way for further research into the relationship between obesity, PR, and non-pharmacological interventions in PR management.

## Introduction

Palindromic rheumatism (PR) represents a distinct stage in the progression toward rheumatoid arthritis (RA), characterized by recurrent joint inflammation episodes of shorter duration and the absence of prolonged joint pain and systemic symptoms associated with RA. PR may advance to RA in a range of 10-66% of patients, contingent upon the quality of follow-up care and treatment [1].

The pathophysiology underlying PR is linked to autoantibodies, specifically rheumatoid factor (RF) and anticitrullinated protein antibodies (ACPA). These autoantibodies target the joints, provoking joint inflammation [1].

The clinical hallmark for diagnosing PR is its relapsing course, characterized by brief episodes of joint inflammation punctuated by pain-free intervals. This diagnosis is substantiated by conducting laboratory tests to detect serum autoantibodies akin to those found in RA [1].

A distinguishing feature of PR, apparent through ultrasound examination, is the presence of extra-capsular inflammation without synovitis. This differs from RA, where synovial inflammation is a primary characteristic [2].

Treatment for PR is empirical. During acute attacks, non-steroidal anti-inflammatory drugs (NSAIDs) can be employed, although their efficacy may vary among patients. Colchicine is effective, particularly in seronegative PR patients. Additionally, antimalarial drugs, such as hydroxychloroquine, have been utilized in PR management [1]. This study reports weight management as a non-pharmacological promising treatment of PR.

## Case presentation

The patient, a 52-year-old Palestinian housewife, experienced a challenging health journey characterized by recurrent episodes of arthritis beginning in 2016. Her symptoms manifested as joint stiffness and swelling initially limited to her wrists but later extended to affect her knees, shoulders, and back. These episodes were debilitating, lasting over an hour and making it nearly impossible for her to perform even the simplest daily tasks. Each episode would persist for several days before spontaneously abating.

Seeking medical intervention, the patient received a diagnosis of RA in 2019, backed by a positive RF test. Consequently, she commenced treatment with disease-modifying anti-rheumatic drugs (DMARDs). Despite the therapeutic regimen, her symptoms remained unrelenting.

In 2022, the patient presented to our hospital, where her medical history was examined meticulously. A distinctive pattern was noticed in her symptoms, their intermittent nature. Furthermore, it was observed that the patient was morbidly obese and displayed no signs of localized joint damage as confirmed by X-ray imaging or other RA-related complications. These distinctive characteristics raised the possibility of PR.

To address both her weight issues and the underlying condition, the patient was approached with a holistic treatment plan. This involved prescribing hydroxychloroquine 200 mg, methotrexate 2.5 mg, and sulfasalazine 500 mg while strongly recommending bariatric surgery as part of the treatment strategy. The patient opted to undergo the surgery, which was successfully performed in 2022. Ten days post-surgery, the patient experienced a remarkable improvement in her symptoms. Additionally, over the next 9 months, her weight had significantly decreased, shedding more than 36 kg. These positive developments further supported the diagnosis of palindromic rheumatism. Currently, she enjoys unrestricted joint mobility and reports only mild discomfort in her fingers after strenuous physical activities.

In response to her markedly improved clinical status (Table 1), it was decided to streamline the patient's medication regimen. She transitioned from taking three different types of medications, amounting to 49 tablets per week, to a single type, hydroxychloroquine 200 mg, with a weekly intake of 7 tablets. This adjustment underscores the potential role of weight management in the management of palindromic rheumatism and offers valuable insights into its clinical course. This patient's case highlights the importance of individualized care in managing unique rheumatological conditions like palindromic rheumatism.

**Table 1:** Comparison table of pre- and post-bariatric surgery-related findings BMI: body mass index; PR: palindromic rheumatism; DMARDs: disease-modifying anti-rheumatic drugs; ESR: erythrocyte sedimentation rate; CRP: C-reactive protein * This includes household responsibilities, cooking and meal preparation, mobility and flexibility

	Pre-bariatric surgery	Post-bariatric surgery
Weight (kg)	120	83
BMI (kg/cm2)	41.5	28.7
PR disease activity	High	Low
Drugs	DMARDs hydroxychloroquine 200 mg, sulfasalazine, methotrexate, and paracetamol	Hydroxychloroquine 200 mg
Daily activity*	Limited daily activities	Free daily activities
Labs	ESR: 33 mm/hr; CRP: 24 mg/L	ESR: 13 mm/hr; CRP<6 mg/L

## Discussion

PR is a rare disease characterized by intermittent, usually monoarticular and asymmetrically distributed, attacks of severe and sudden joint swelling, gradually peaking pain, and erythema involving most commonly the wrists, shoulders, knees, ankles, and small joints of the hand [3]. Each attack lasts a few hours to days, leaving no articular damage. The clinical profile of PR is characterized by the absence of fever, negative radiological tests, and increased acute phase reactants during attacks, which become normal in symptom-free intervals [4]. Due to the absence of clear diagnostic criteria or accepted definitions, PR diagnosis is challenging and may take extended time periods and clinical encounters [1]. In our case, the diagnosis was made after three years. All this time our patient complained of morning pain and stiffness in her wrists, ankles, shoulders, and back lasting a few hours. 

The exact etiology and pathogenesis are not well-established. However, the roles of autoimmunity are believed to play a major role in the development of PR. Many PR patients show similar serum autoantibody to that of RA, which suggests PR is a pre-stage of chronic rheumatic disease. Patients with PR often go on to develop RA [1,5]. 

This uncertainty of PR pathogenesis provokes challenges in its management. However, rising evidence states that PR can be targeted by three distinct treatment facets: non-steroidal anti-inflammatory drugs (NSAIDs), analgesics, and systemic corticosteroids are used to treat flares. DMARDs are available in three distinct forms to reduce relapses and prevent the disease progression to RA: conventional DMARDs (hydroxychloroquine, methotrexate, sulfasalazine, etc.), biological DMARDs (rituximab), and other DMARDs (D-penicillamine and azathioprine) [2]. In our case, the patient was prescribed hydroxychloroquine, methotrexate, sulfasalazine, and paracetamol. 

Our case demonstrated a potential association between obesity and PR. Our patient suffered from being overweight with a BMI of 41.5, leading to her decision to undergo bariatric surgery three years after her diagnosis, after which she lost 36 kg. This significant weight loss drastically changed our patient's status, marking better progress in her condition. Attacks were reduced significantly leading her doctor to reduce the drug regimen only to hydroxychloroquine. Following the surgery, a sequential reduction in erythrocyte sedimentation rate (ESR) levels over the course of 9 months was observed; ESR levels have returned within the normal range. 

Obesity may be linked to chronic systemic inflammation since biological mechanisms of inflammation are present in adipose tissue. Moreover, adipokines have been shown to play a significant role as mediators of inflammation and immune responses, which are implicated in rheumatic inflammatory diseases. Several well-conducted studies reported a large effect of obesity on RA [6]. A study suggested that obesity and increased adiposity are linked to a decreased tendency of RA patients to achieve remission, furthermore, the response to disease-modifying agents and anti-TNF alpha antibodies have been altered in obese people as they were shown to have decreased response [7]. According to a study that demonstrated obesity as a risk factor for RA, patients with RA who underwent bariatric surgery and lost a significant amount of weight had better responses to RA medications [8]. In support, another study illustrated the effect of bariatric surgery on metabolism, which in turn decreased the use of immunosuppressive medications in patients with rheumatic diseases [9]. However, no studies were conducted to examine obesity's effect on PR.

This case establishes bariatric surgery as a non-pharmacological promising treatment of PR. Obesity has been linked to autoinflammatory diseases, which foster the relationship between weight loss and PR management and open new horizons of research and investigation [6]. 

## Conclusions

This case underscores the intricacies of palindromic rheumatism (PR), shedding light on its distinct clinical characteristics and the potential interplay with obesity. PR, often a precursor to rheumatoid arthritis (RA), poses diagnostic challenges due to its intermittent symptom patterns. This case highlights the importance of meticulous clinical evaluation in distinguishing PR from RA, ensuring appropriate management and tailored treatments. Unlike RA, PR attacks come in a relapsing/remitting course and leave no joint damage.

Furthermore, the significant improvement in our patient’s condition following bariatric surgery underscores the potential role of weight management in PR therapy. While more research is needed to unravel the relationship between obesity and autoimmune diseases, this case offers a valuable perspective on non-pharmacological interventions in PR management, emphasizing the importance of personalized care in addressing this complex rheumatological condition.
